# Effect of Tumor-Pixel Positioning on the Variability of SUV Measurements in PET Images

**DOI:** 10.22038/AOJNMB.2022.61623.1434

**Published:** 2023

**Authors:** Koji Itagaki, Katsuhiko Mitsumoto, Masaaki Kajisako, Maki Shioji, Shigeto Kawase

**Affiliations:** Division of Clinical Radiology Service, Kyoto University Hospital, Kyoto, Japan

**Keywords:** PET/MR, SUV measurement Reproducibility

## Abstract

**Objective(s):**

The aim of this study was to investigate the effect on standardized uptake value (SUV) measurement variability of the positional relationship between objects of different sizes and the pixel of a positron emission tomography (PET) image.

**Methods:**

We used a NEMA IEC body phantom comprising six spheres with diameters of 10, 13, 17, 22, 28, and 37 mm. The phantom was filled with ^18^F solution and contained target-to-background ratios (TBRs) of 2, 4, and 8. The PET data were acquired for 30 min using a SIGNA PET/MR scanner. The PET images were reconstructed with the ordered subsets expectation maximization (OSEM) algorithm with and without point-spread function (PSF) correction (OSEM + PSF + Filter and OSEM + Filter, respectively). A Gaussian filter of 4 mm full width at half maximum was applied in all reconstructions, except for one model (OSEM + PSF + no Filter). The matrix sizes were 128×128, 192×192, 256×256 and 384×384. Reconstruction was performed by shifting the reconstruction center position by 1 mm in the range 0 to 3 mm in the upward or rightward direction for each parameter. For all reconstructed images, the SUV_max_ of each hot sphere was measured. To investigate the resulting variation in the SUV_max_, the coefficient of variation (CV) of each SUV_max_ was calculated.

**Results:**

The CV of the SUV_max_ increased as the matrix size and the diameter of the hot sphere decreased in all reconstruction settings. With PSF correction, the CV of SUV_max_ increased as the TBR increased except when the TBR was 2. The CV of the SUV_max_ measured in the OSEM + PSF + no Filter images were larger than those measured in the OSEM + PSF + Filter images. The amount of this increase was higher for smaller spheres and larger matrix sizes and was independent of TBR.

**Conclusions:**

Shifting the reconstruction center position of the PET image causes variability in SUV_max_ measurements. To reduce the variability of SUV measurements, it is necessary to use sufficient matrix sizes to satisfy sampling criterion and appropriate filters.

## Introduction

 Malignant tumors are often detected and staged using ^18^F-fluoro-2-deoxy-D-glucose (^18^F-FDG) positron emission tomography (PET) (1). In FDG-PET, visual assessment is widely used to diagnose malignancy, but semi-quantitative analysis using a standardized uptake value (SUV) is also used (2, 3). SUVs are also an important tool for monitoring the response to therapy of various malignant tumors (4). 

 However, many biological and technological factors affect SUV measurements (5). SUV_max _has higher interobserver reproducibility, which is important for multicenter studies evaluating the usefulness of ^18^F-FDG PET for treatment monitoring (6), but because SUV_max_ is sensitive to noise, the within-patient SD for SUV_max_ is higher in noisy conditions than that for SUV_mean_ or SUV_peak_ (5,7,8,9). However, SUV_max_ is still widely used because it can be easily measured from any workstation and avoid inclusion of necrotic or other non-tumor elements (10-12). 

 The mean absolute percentage difference between successive SUV_max_ measurements has been reported to be 13%±12 (10) and 11.3%±8.0 (13). Another group reported that the within-patient SD for SUV_max _varied from 10.7% to 12.8% (14). In addition, the within-patient SD for SUV_max_ including only the effects of image noise was reported to be 5.6%±0.9 (7). 

 Recent advancements in the hardware and software of PET devices, such as reductions in detector size, increases in signal-to-noise ratio using time-of-flight (TOF) techniques, and improvements in spatial resolution using point-spread function (PSF) reconstruction have improved the detectability of small lesions (15). 

 In addition, it has been reported that the detectability of small lesions is improved with long acquisition durations (8), which should also enable small lesions to be clearly delineated in PET/MR generally. SUV measurement in small lesions is, however, often affected by the partial volume effect (PVE) (9).

 PVE in PET image mainly results from the finite spatial resolution of the imaging system and image sampling, and occurs whenever the tumor size is less than three times the full width half maximum (FWHM) of the spatial resolution (8). The spatial resolution in PET images is limited by the physical size of detector, positron range, radius of the tomograph detector ring, and reconstruction method (16). As for the reconstruction methods, increasing the number of iterations improves the spatial resolution because higher frequencies can be recovered (17), which may improve the detection of small lesions, but also increase image noise (18). In PET/MR, a longer acquisition duration reduces image noise and enables the use of reconstruction methods with higher spatial resolutions. By contrast, in image sampling, large pixels are more likely to underestimate the SUV. Moreover, it has been reported that there is variability in SUV_max_ depending on the positional relationship between the object and the pixel of the PET image (5). Maebatake et al. reported that the repeatability of a small hot spot was affected by the positional relationship between the subject and pixels (19). However, the variability of SUV measurements due to the positional relation-ship may also depend on the size of the object as well as the finite spatial resolution of the imaging system.

 The aim of the present study was to investigate the effect of the positional relationship between objects of different sizes and the pixels of the PET image on the variability of SUV measurements using a National Electrical Manufacturers Association (NEMA) International Electrotechnical Commission

(IEC) body phantom.

## Methods

***Phantom***

 We used a NEMA IEC body phantom comprising six spheres with diameters of 10, 13, 17, 22, 28, and 37 mm. All spheres and the background of the phantom were filled with ^18^F solution. The phantom contained target-to-background ratios (TBRs) of 2, 4, and 8 on a background of 5.3 kBq/mL of radioactivity concentration, according to the Japanese guidelines for oncological ^18^F-FDG PET/CT data acquisition (20).

***Data Acquisition***

 PET data were acquired using a PET/MR scanner (SIGNA PET/MR; GE Healthcare) with silicon photomultipliers (SiPMs) and TOF-PET capability. This PET scanner comprised five rings with a total of 20,160 lutetium-based scintillation crystals with dimensions of 4.0×5.3×25 mm^3^. Sinogram plane had 224 (radial coordinate) ×357 (azimuthal angle) bins, as no transverse mashing was applied. This system had axial and transaxial fields of view of 25 and 60 cm, respectively. The coincidence time window was 4.57 ns. The TOF timing resolution is 386 ps. The spatial resolutions at 1 and 10 cm from the center of the field of view were 4.2 and 5.2 mm in FWHM, respectively. The PET data were acquired in 3-dimensional list mode for 30 min.

***Image reconstruction***

**Figure 1:**
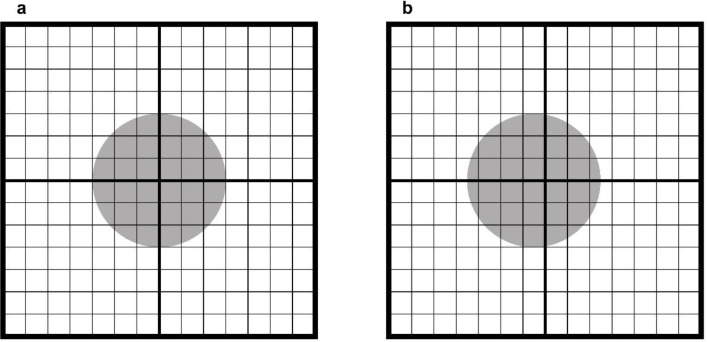
Reconstructed image without (**a**) and with (**b**) a rightward shift in the reconstruction center position. The grid represents the image matrix, and the gray sphere represents the hot sphere

 PET images were reconstructed using a 3-dimensional ordered subsets expectation maximization (OSEM) algorithm with PSF correction (OSEM + PSF + Filter) using four iterations and 16 subsets. A Gaussian filter of 4 mm FWHM was applied in the reconstruction. The reconstruction parameters were determined according to the clinical setting for PET/MR examinations with an acquisition time of fifteen to twenty minutes. To investigate the effect of matrix size on the variability of SUV measurements due to the positional relation-ship, we reconstructed PET images with matrix size of 128×128, 192×192, 256×256, and 384×384. Pixel sizes were 4.7×4.7, 3.1×3.1, 2.3×2.3, and 1.6×1.6 mm, respectively. Although large pixel sizes may violate the sampling criterion, the commercial reconstruction software allows the selection of matrix size of 128×128 or 192×192, which may be used. In addition, to evaluate the effect of PSF correction on its variability, we reconstructed PET images without PSF correction (OSEM + Filter). No post-smoothing was applied in one OSEM+PSF model (OSEM + PSF + no Filter) to evaluate the effect of the post-smoothing filter. All PET images were reconstructed with TOF information. The PET image slice thickness was 2.78 mm. The scatter correction was conducted using single scatter simulation, and the attenuation correction was performed using CT measurement-based attenuation templates. To vary the relationship between the subject and the pixel of the PET image, reconstruction was performed while shifting the reconstruction center position in intervals of 1 mm from 0 to 3 mm in the upward or rightward direction for each parameter (Figure 1). Therefore, for each reconstruction parameter, 16 sets of images were reconstructed.

***Data analysis***

 All PET datasets were analyzed using an Advantage Workstation (GE Healthcare). For all reconstructed images, the SUV_max_ of each hot sphere was determined from a spherical volume of interest with a diameter large enough to include each hot sphere.

 To investigate the variation in the SUV_max_ of each hot sphere when the reconstruction center position was shifted, the coefficient of variation (CV) of SUV_max_ was calculated using the following equation**.**

$\mathrm{CV}=\frac{\sigma }{\overset{\text{®}}{{\mathrm{SUV}}_{\max}}}\times 100(\%)$

 Here, σ and$\overset{\text{®}}{{\mathrm{SUV}}_{\max}}$ are respectively the standard deviation and mean of the SUV_max _of each measured hot sphere.

## Results

**Figure 2:**
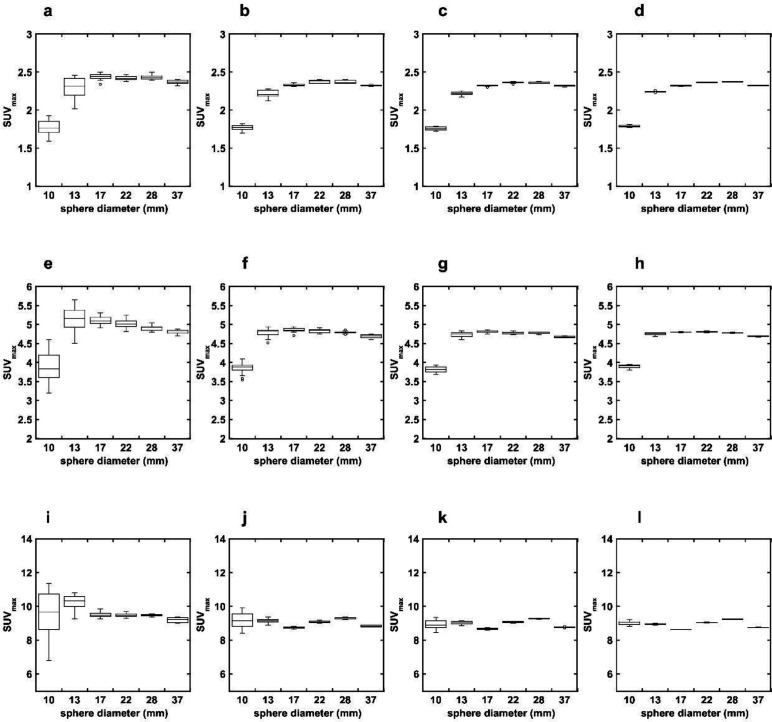
Relationship between sphere size and SUV_max_ at different matrix sizes in the OSEM + PSF + Filter images. The matrix sizes are 128 (**a**, **e**, **i**), 192 (**b**, **f**, **j**), 256 (**c**, **g**, **k**), and 384 (**d**, **h**, **l**) at a TBR of 2 (upper row), 4 (middle row), and 8 (lower row). The ∆SUV_max _was higher for smaller matrix sizes and smaller diameters of the hot sphere

 The SUV_max_ of each hot sphere measured in the OSEM + PSF + Filter images with different matrix sizes and shifted reconstruction center positions at each TBR are shown in Figure 2. The difference between the maximum and minimum SUV_max_ (ΔSUV_max_) of the 10-mm hot sphere increased as the matrix size decreased. The CV of the SUV_max_ of each hot sphere measured in the OSEM + PSF + Filter images with different matrix sizes at each TBR are shown in Table 1. The CV of increased as matrix size decreased, sphere diameter decreased, and TBR increased. The CV of the 10-mm hot sphere were 13.82%, 5.14%, 2.91%, and 1.18% at a TBR of 8 for matrix sizes of 128, 192, 256, and 384, respectively.

**Table 1:** CV of SUV_max_ in the OSEM + PSF + Filter images for different matrix sizes at each TBR

**TBR**	**Spere diameter (mm)**	**Matrix size**			
		**128**	**192**	**256**	**384**
2	10	5.55	1.85	1.27	0.57
	13	5.41	2.15	1.01	0.35
	17	1.56	0.63	0.36	0.34
	22	0.96	0.77	0.41	0.20
	28	1.19	0.78	0.47	0.21
	37	0.95	0.42	0.40	0.21
4	10	10.13	4.13	2.15	1.02
	13	5.91	2.49	1.31	0.67
	17	2.31	1.16	0.59	0.25
	22	2.18	1.07	0.63	0.33
	28	1.29	0.64	0.48	0.19
	37	1.00	0.97	0.38	0.25
8	10	13.82	5.14	2.91	1.18
	13	4.26	1.41	0.94	0.29
	17	1.57	0.56	0.52	0.13
	22	1.07	0.64	0.43	0.16
	28	0.50	0.55	0.15	0.13
	37	1.57	0.59	0.24	0.13

**Figure 3:**
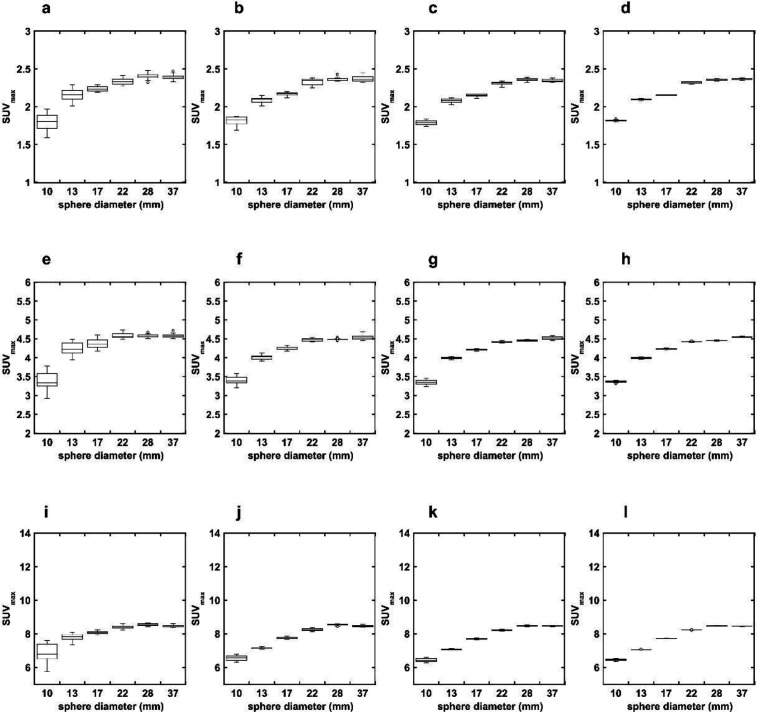
Relationship between sphere size and SUV_max_ at different matrix sizes in the OSEM + Filter images (without PSF). The matrix sizes are 128 (**a**, **e**, **i**), 192 (**b**, **f**, **j**), 256 (**c**, **g**, **k**), and 384 (**d**, **h**, **l**) at a TBR of 2 (upper row), 4 (middle row), and 8 (lower row). The ΔSUV_max_ was lower than in the OSEM + PSF + Filter images (Figure 2) with smaller diameters of the hot sphere at a TBR of 4 and 8

Figure 3 shows the SUV_max_ of each hot sphere measured in the OSEM + Filter images with different matrix sizes and shifted reconstruction center positions at each TBR. The SUV_max_ of the 10-mm hot sphere measured in OSEM + Filter images was smaller than that measured in the OSEM + PSF + Filter images at TBRs of 4 and 8. The CV of the SUV_max _of each hot sphere measured in the OSEM + Filter images with different matrix sizes at each TBR are shown in Table 2. The CV of SUV_max _increased as matrix size and sphere decreased, but was independent of TBR. The CV of the 10-mm hot sphere were 7.83%, 2.20%, 1.67%, and 0.66% at a TBR of 8 for matrix sizes of 128, 192, 256, and 384, respectively.

**Table 2:** CV of SUV_max_ in the OSEM + Filter images (without PSF) for different matrix sizes at each TBR

**TBR**	**Spere diameter (mm)**	**Matrix size**			
		**128**	**192**	**256**	**384**
2	10	5.67	2.92	1.76	0.57
	13	3.73	1.69	1.24	0.48
	17	1.42	1.00	0.82	0.23
	22	1.77	1.82	0.94	0.39
	28	1.62	1.02	0.78	0.41
	37	1.46	1.62	0.80	0.39
4	10	7.57	3.18	1.92	0.81
	13	3.82	1.47	0.59	0.42
	17	2.95	0.95	0.39	0.32
	22	1.67	0.87	0.38	0.24
	28	1.04	0.46	0.36	0.18
	37	1.24	1.34	0.83	0.26
8	10	7.83	2.20	1.67	0.66
	13	2.53	0.50	0.31	0.21
	17	0.98	0.59	0.31	0.15
	22	1.21	0.88	0.45	0.14
	28	0.76	0.61	0.40	0.18
	37	0.71	0.60	0.26	0.12

**Figure 4:**
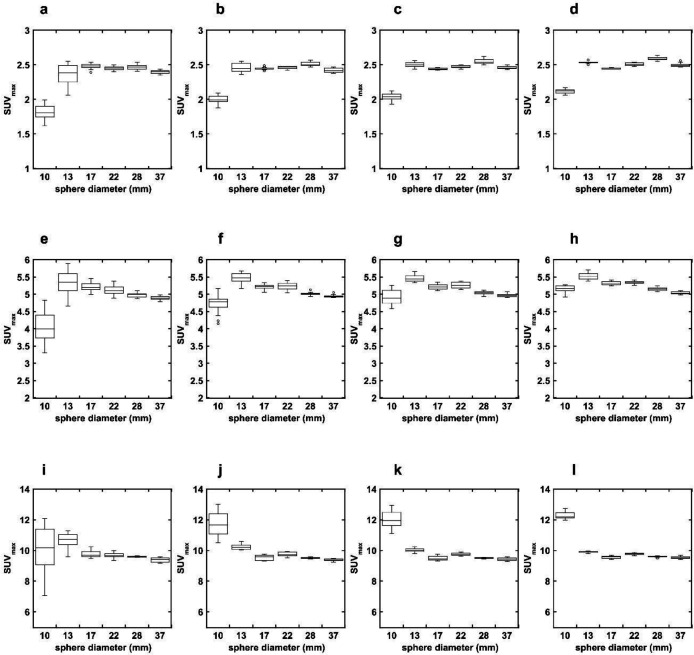
Relationship between sphere size and SUV_max_ at different matrix sizes in the OSEM + PSF + no Filter images. The matrix sizes are 128 (**a**,** e**, **i**), 192 (**b**, **f**, **j**), 256 (**c**, **g**, **k**), and 384 (**d**, **h**, **l**) at a TBR of 2 (upper row), 4 (middle row), and 8 (lower row). The ΔSUV_max_ was higher than in the OSEM + PSF + Filter images (Figure 2)

Figure 4 shows the SUV_max _of each hot sphere measured in the OSEM + PSF + no Filter images with different matrix sizes and shifted reconstruction center positions at each TBR. The CV of SUV_max_ of each hot sphere measured in the OSEM + PSF + no Filter images with different matrix sizes at each TBR are shown in Table 3. These results are larger than those of the OSEM + PSF + Filter images. The degree of increase in CV was higher for smaller spheres and larger matrix sizes, but was independent of TBR. The CV of the 10-mm hot sphere were 14.39%, 6.89%, 4.52%, and 1.83% at a TBR of 8 for matrix sizes of 128, 192, 256, and 384, respectively.

**Table 3:** CV of SUV_max_ in the OSEM + PSF + no Filter images for different matrix sizes at each TBR

**TBR**	**Spere diameter (mm)**	**Matrix size**			
		**128**	**192**	**256**	**384**
2	10	5.93	2.76	2.49	1.45
	13	5.77	2.50	1.27	0.72
	17	1.52	0.82	0.50	0.23
	22	1.01	0.81	0.77	0.79
	28	1.43	1.19	1.44	0.99
	37	1.04	1.29	0.86	1.02
4	10	10.70	6.05	4.21	1.81
	13	6.14	2.93	1.77	1.64
	17	2.49	1.24	1.42	0.92
	22	2.46	1.96	1.67	0.82
	28	1.29	0.89	0.93	0.97
	37	1.11	0.87	0.91	0.76
8	10	14.39	6.89	4.52	1.83
	13	4.45	1.54	1.12	0.40
	17	2.13	1.75	1.57	0.84
	22	1.62	1.32	0.84	0.67
	28	0.34	0.36	0.33	0.46
	37	1.50	0.71	1.03	0.67

## Discussion

 In this study, we investigated the effect of the positional relationship between an object and a pixel of a PET image on the variability of SUV measurements. To change the relationship between the subject and the pixels of PET images, we reconstructed PET images by shifting the reconstruction center position by 1 mm in the upward or rightward direction.

 Shifting this reconstruction center position causes variability in SUV_max_ measurements. The degree of variation depends on the reconstruction conditions (matrix size, post-smoothing filter, and PSF correction) as well as TBR. Biological factors, scanner variability, and reconstruction parameters will affect SUV measurements (5). In this study, the same emission data were used for each TBR to minimize the effects of phantom preparation and placement accuracy. We also acquired the data over a long duration to reduce the effect of statistical noise. Therefore, the main cause of the variability in SUV_max_ in this study appears to be PVE related to image sampling. The CV of SUV_max_ increased as matrix size and the diameter of the hot sphere decreased because of the increase in PVE related to image sampling. 

**Figure 5:**
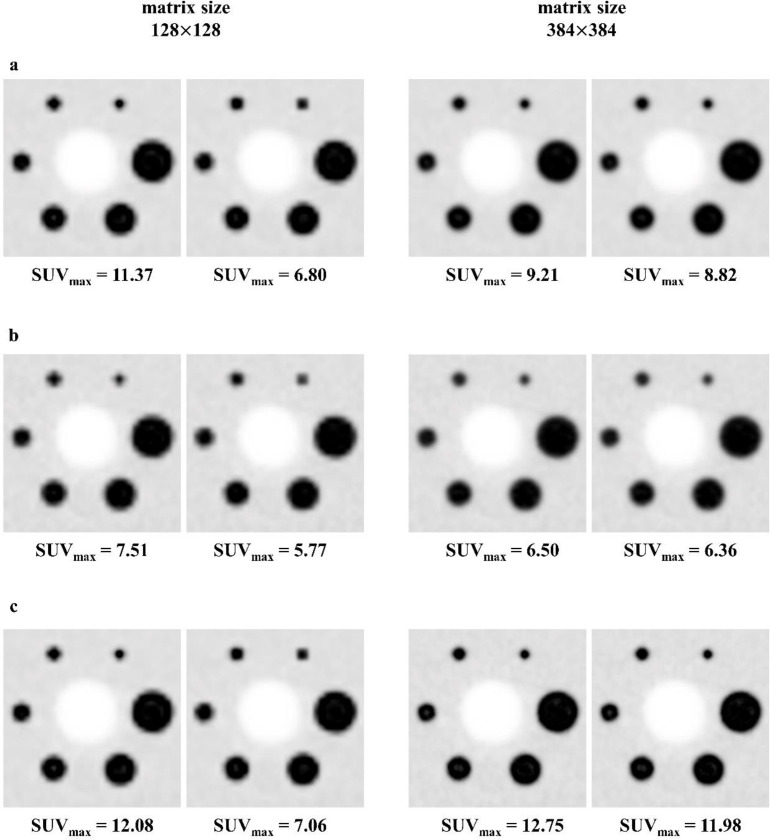
PET images reconstructed at different reconstruction center position. Matrix sizes are 128×128 and 384×384 at a TBR of 8. The images reconstructed with OSEM + PSF + Filter (**a**), OSEM + Filter (without PSF) (**b**), OSEM + PSF + no Filter (**c**). The values at the bottom of the images are the maximum and minimum SUV_max_ of the 10-mm hot sphere for each reconstruction settings

 The measurement of small lesions is particularly affected by PVE, and PET counts are underestimated when the image of the hot sphere lies between certain pixels (Figure 5) (8). Another reason for the increase of the CV of SUV_max_ might be due to the change in the influence of spill-out and　aliasing depending on of the positional relationship between hot spheres and a pixel. With larger pixel sizes, their effect might be significant (8).

 With PSF correction, the CV of SUV_max_ increased with increases in TBR as well as with decreases in matrix size and the diameter of the hot sphere. One cause of the increase in variability of SUV_max_ with PSF correction seems to be edge artifacts due to PSF correction. For small spheres, this artifact was observed as a sharp peak at the center of spheres that increased the PVE related to image sampling (16,17). At lower TBRs, the edge artifact was less likely to appear, so the CV of SUV_max_ did not increase as much (17).

 In addition, for the same TBR, the CV of SUV_max _decreased when no PSF correction was used, except when the TBR was 2. It has been reported that the variability in SUV measurement increases with PSF correction (16). Munk et al. reported that the lumpy noise caused by PSF correction decreases the reproducibility of SUV measurement (21). 

 Moreover, PET images without PSF correction are more blurred than those with PSF correction, resulting in reduced PVE and decreased variability. At a TBR of 2, two factors can be considered the reasons the CV of SUV_max_ does not decrease when no PSF correction is used.

 The first factor is the improvement in spatial resolution obtained by PSF correction, which is dependent on concentration. At a low TBR, the resolution is not as improved as it is at high TBR, so PET images taken at low TBRs are blurrier (17, 22, 23). Therefore, the results were less affected by PVE related to image sampling. The second factor is the improvement in signal-to-noise ratio caused by PSF correction (24). In the hot sphere at a low TBR, image noise is relatively high due to the low concentration of radioactivity, and the CV of SUV_max_ increased, especially when PSF correction was not used. Without PSF correction, the higher CV of SUV_max_ of the 37 mm sphere, which should have been less sensitive to PVE, indicates that image noise is one factor affecting the variation at a TBR of 2. Note that the use of PSF correction has been reported to improve the detectability of small lesions (25, 26). The trade-off between lesion detectability and the accuracy of SUV measurement must be considered when determining the reconstruction parameters.

 The CV of the SUV_max_ measured in the OSEM + PSF + no Filter images was larger than that measured in the OSEM + PSF + Filter images. 

 The degree of increase in CV of SUV_max_ was higher for smaller spheres and larger matrix sizes but independent of TBR. Without post-smoothing, the measurement of the PET counts was more affected by PVE related to image sampling because of the sharper profile of the hot sphere, resulting in a larger variation in small spheres when the reconstruction center position was shifted.

 For large matrix sizes, SUV_max_ is higher because of increased image noise. This can be suppressed by applying a post-smoothing filter (27, 28). Recently, several studies reported that the detectability of small lesions improves with the use of larger matrix sizes (15, 25, 29). Another study reported that reconstruction parameters affect the SUV measurement of small lesions (26). When evaluating small lesion uptake, it is necessary to increase the matrix size sufficiently to reduce the variability caused by PVE related to image sampling and to use an optimal post-smoothing filter to prevent overestimation of SUV_max_ due to image noise. In this study, we applied the fixed filter size at 4 mm FWHM to evaluate the effect of the post-smoothing filter. However, in clinical practice, it is necessary to use the optimal filter for the pixel size.

 Several groups have evaluated the reproducibility of SUV measurements (7, 10, 13, 14). They reported that the CV of SUV_max_ was approximately 8.0%–13%. Those studies did not mention the tumor size, but in the present study, the CV of SUV_max_ reached 14.39% using the same phantom data and reconstruction parameters. Therefore, the variability in SUV_max_ caused by shifting the reconstruction center position is considered to be an important factor in SUV measurement.

 The present study has several limitations. First, the target objects we measured were spherical and homogeneous. PVE depends on the shape and homogeneity of the tumor (8). A simulation study may address this limitation. Second, the PSF correction is position dependent, and the degree of variation may change depending on the position of the hot sphere. In addition, a long acquisition time of 30 minutes was used to exclude the effect of statistical noise on the variability of SUV measurements in this study, but further evaluation with actual clinical acquisition time is necessary. Finally, we evaluated the variability of SUV_max_ due to the positional relationship, but SUV_mean_ and SUV_peak_ are also an important tool for monitoring the response to therapy of malignant tumors and needs to be evaluated in the future (30, 31). 

## Conclusion

 Shifting the reconstruction center position in PET image reconstruction causes variability in SUV_max_ measurements. To reduce the variability of SUV measurements, it is necessary to use sufficient matrix sizes to satisfy sampling criterion and appropriate filters.

## Conflict of interest

 The authors declare that they have no conficts of interest.
